# Achilles tendon morpho-mechanical parameters are related to triceps surae motor unit firing properties

**DOI:** 10.1152/jn.00391.2023

**Published:** 2024-09-04

**Authors:** Ignacio Contreras-Hernandez, Michail Arvanitidis, Deborah Falla, Francesco Negro, Eduardo Martinez-Valdes

**Affiliations:** ^1^Centre of Precision Rehabilitation for Spinal Pain (CPR Spine), School of Sport, Exercise and Rehabilitation Sciences, College of Life and Environmental Sciences, University of Birmingham, Birmingham, United Kingdom; ^2^Department of Clinical and Experimental Sciences, Università degli Studi di Brescia, Brescia, Italy

**Keywords:** Achilles tendon, HD-sEMG, mechanical properties, morphological properties, motor unit

## Abstract

Recent studies combining high-density surface electromyography (HD-sEMG) and ultrasound imaging have yielded valuable insights into the relationship between motor unit activity and muscle contractile properties. However, limited evidence exists on the relationship between motor unit firing properties and tendon morpho-mechanical properties. This study aimed to determine the relationship between triceps surae motor unit firing properties and the morpho-mechanical properties of the Achilles tendon (AT). Motor unit firing properties [i.e. mean discharge rate (DR) and coefficient of variation of the interspike interval (COVisi)] and motor unit firing-torque relationships [cross-correlation between cumulative spike train (CST) and torque, and the delay between motor unit firing and torque production (neuromechanical delay)] of the medial gastrocnemius (MG), lateral gastrocnemius (LG), and soleus (SO) muscles were assessed using HD-sEMG during isometric plantarflexion contractions at 10% and 40% of maximal voluntary contraction (MVC). The morpho-mechanical properties of the AT (i.e. length, thickness, cross-sectional area, and resting stiffness) were determined using B-mode ultrasonography and shear-wave elastography. Multiple linear regression analysis showed that at 10% MVC, the DR of the triceps surae muscles explained 41.7% of the variance in resting AT stiffness. In addition, at 10% MVC, COV_isi_ SO predicted 30.4% of the variance in AT length. At 40% MVC, COV_isi_ MG and COV_isi_ SO explained 48.7% of the variance in AT length. Motor unit-torque relationships were not associated with any morpho-mechanical parameter. This study provides novel evidence of a contraction intensity-dependent relationship between motor unit firing parameters of the triceps surae muscle and the morpho-mechanical properties of the AT.

**NEW & NOTEWORTHY** By employing HD-sEMG, conventional B-mode ultrasonography, and shear-wave elastography, we showed that the resting stiffness of the Achilles tendon is related to mean discharge rate of triceps surae motor units during low-force isometric plantarflexion contractions, providing relevant information about the complex interaction between rate coding and the muscle-tendon unit.

## Graphical abstract

**Figure:**
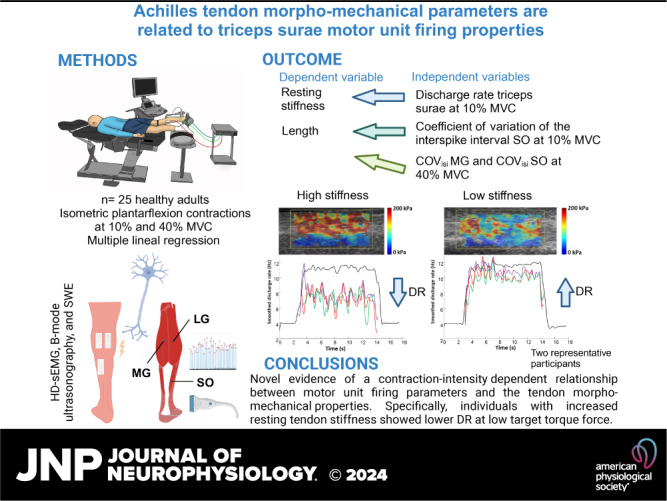

## INTRODUCTION

Human movement emerges from the interplay between descending output from the central nervous system (CNS), sensory input from the body and environment, muscle dynamics, and whole body dynamics (1). Thus, the CNS plans and sends motor commands to the muscle fibers via motoneurons (2) that translate these neural commands into forces (3), which are then transmitted via connective tissue to the skeletal system to generate movement (4). Within this framework, the muscle-tendon unit can be considered as a functional component of human movement capable of working as a motor, damper, and spring to exert, dissipate or store, and release energy (5, 6). These complex functions are possible by serial and parallel coupling of active force-generating tissues and passive force-transmitting tissues and by using the ability to shift energy between active and passive components (7). Passive elastic components include tendons and aponeurosis, which transmit force in series with the active force generated by the muscle’s fibers (8). It has been long recognized that the ability of a muscle to control the length changes of its fibers relative to the stretching of its tendon during a contraction is influenced by its architecture and the physiological characteristics of its fibers (8). However, the interplay between neural modulation of the muscle and the morpho-mechanical properties of the tendon has received less attention.

In human locomotion, elastic energy, defined as the potential energy stored within the elastic tissues of the muscle-tendon units, is efficiently stored and released in the lower limb during the contact and push-off phases, respectively (9, 10). However, this adaptability requires that the amount of elastic energy stored should be modulated by muscular contraction (9). Based on this, several studies have used the triceps surae muscle to investigate how elastic energy can be stored and released efficiently (11, 12). The triceps surae plays a crucial role in human plantarflexion, which is primarily accomplished by the medial gastrocnemius (MG), lateral gastrocnemius (LG), and soleus (SO) muscles (13). Despite being agonist muscles that share the same common distal tendon, these muscles have anatomical, neurophysiological, and functional differences suggesting diverse functional roles (13). These functional roles are associated with distinctive motor unit firing rate properties between muscles during different tasks (13, 14). In parallel, the Achilles tendon (AT) has been investigated extensively due to its critical role in lower limb biomechanics (7, 15). The AT is the largest, thickest, and strongest tendon of the human body (16–18), and it transmits forces generated by the strongest ankle plantar flexors (19). This muscle-tendon complex crosses and acts on the knee, ankle, and subtalar joints (17). Studies investigating the features of the AT include morphological properties [i.e., length, thickness, cross-sectional area (CSA), and width] (20, 21), and mechanical characteristics (i.e., Young’s modulus, stress, strain, hysteresis, and tensile rupture stress) (10, 20) or both. In vivo methodologies to determine the mechanical properties of the AT are becoming more frequently used due to their ability to assess the mechanical behavior of the AT during various activities (22–25). During the past few years, shear-wave elastography (SWE) has been increasingly used to study the mechanical properties of tendons (26). SWE has the advantage of being able to measure the speed of shear stress wave propagation, allowing the calculation of Young’s modulus (i.e., tendon stiffness) (27).

Recent studies combining ultrasound imaging and electromyography techniques have assessed the mechanisms responsible for converting neural activity into muscle contractions (28–30). These techniques have provided a more comprehensive description of the events underlying the generation of muscle force (31). For instance, they have revealed the relationship between muscle activation and fascicle length during different postural conditions (30), the spatiotemporal associations between electrical and mechanical properties of active motor units (31), or the relationship between motor unit firing properties, fascicle length, and torque (32). However, there is limited evidence of the relationship between motor unit firing properties and the morpho-mechanical properties of tendons. Studies investigating the effect of static-stretch interventions on the triceps surae have shed light on this relationship (33–35). For example, Mazzo et al. (35) have shown that after a static-stretch intervention on the triceps surae, there is an increase in motor unit discharge rate and a decrease in motor unit recruitment threshold at low forces (10% of the maximum). Furthermore, Trajano et al. (34) found similar increases in soleus muscle discharge rate at low forces following calf-muscle stretching. It is possible that stretching-induced changes in the morpho-mechanical properties of the AT were related to changes in the motor unit discharge rate of the triceps surae muscles, however, this was not assessed in those studies.

We aimed to determine the relationship between triceps surae motor unit firing properties [i.e., mean discharge rate (DR) and discharge rate variability (estimated by the coefficient of variation of the interspike interval (COV_isi_)] and the morpho-mechanical properties of the AT. In addition, we assessed motor unit firing-torque relationships [i.e., cross-correlation coefficient between cumulative spike train (CST) and torque, and neuromechanical delay (NMD)] and their association with morpho-mechanical properties of the AT. We assessed which triceps surae motor unit discharge properties or motor unit firing-torque relationships would explain most of the variance in tendon length, thickness, CSA, and the estimated resting stiffness via multiple regression analysis. As force generation is the result of the relationship between the neural drive received by muscles (i.e., motor unit firing rate and recruitment) and muscle-tendon unit behavior, we hypothesized that there is a relationship between DR and the mechanical properties of the AT; thus, we expect that individuals with greater resting AT stiffness will show lower DR.

## MATERIALS AND METHODS

### Participants

Twenty-five healthy (17 males, 8 females, 28.60 ± 3.92 yr, 74.00 ± 11.57 kg, 171.10 ± 9.22 cm) participants were recruited from the University of Birmingham staff/student population and the local community via leaflets, e-mail, and social media. Men or women aged 18 to 55 yr old were recruited; this age range was selected to minimize aging-related changes of the tendon, as previous studies have found lower stiffness and Young’s modulus of the AT in older than in younger populations (36). Inclusion criteria include confirmation of a healthy AT determined by an experienced physiotherapist through physical examination and ultrasound imaging. Ultrasound imaging included assessing normal tendon thickness (no focal or diffuse thickening) and echoic pattern (no focal hypoechoic and hyperechoic areas within the tendon) (37). Exclusion criteria included the following: *1*) systemic or inflammatory conditions including rheumatic, neuromuscular disorders, and malignancy, *2*) current or previous history of chronic respiratory, neurological, or cardiovascular diseases, *3*) history of Achilles tendinopathy or lower limb surgery, and *4*) pain/injury in the lower limbs within the previous 6 mo.

### Study Design

This cross-sectional study was conducted from October 2021 to December 2022 at a laboratory within the Centre of Precision Rehabilitation for Spinal Pain (CPR Spine), University of Birmingham, UK. The Science, Technology, Engineering and Mathematics Ethical Review Committee, University of Birmingham, UK, approved the study (ERN_20-0604A). The study was conducted according to the Declaration of Helsinki and all participants provided written informed consent before participation. The guideline for Strengthening the Reporting of Observational Studies in Epidemiology (STROBE) was used to facilitate reporting (38).

Participants visited the laboratory once for the experimental session (2.5 h) and were asked to avoid any strenuous physical activity 24 h before testing. The assessed leg was randomized across participants. A subgroup of participants (3 males, 3 females, 27.17 ± 4.49 yr, 68.42 ± 7.17 kg, 167.33 ± 7.22 cm) visited the laboratory twice (1 wk apart) to confirm the intratester reliability of b-mode ultrasound and SWE measurements. During this period, participants were instructed to maintain their level of physical activity and avoid any strenuous physical activity 24 h before testing.

### Experimental Setup and Tasks

**Figure 1.**
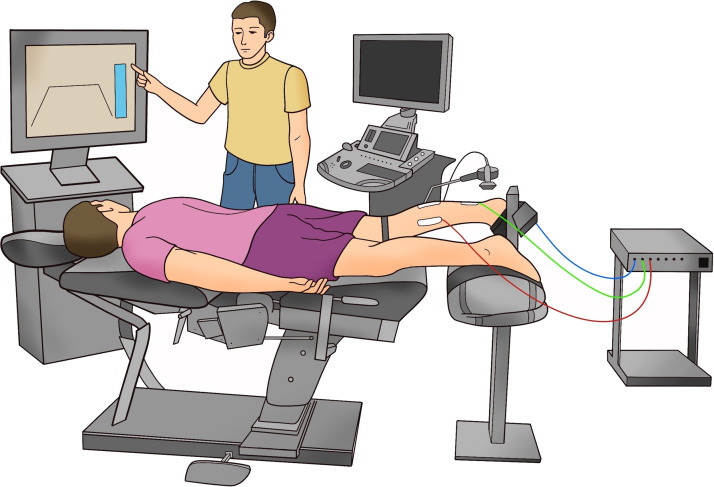
Representation of the experimental setup. The participant is shown in a prone position on an isokinetic dynamometer, with the right foot secured to the ankle attachment. Three electrode grids are placed on the triceps surae muscles and connected to the signal amplifier. A researcher is indicating the visual feedback that the participant must follow during the isometric plantarflexion contraction assessments. In addition, the ultrasound equipment used to assess tendon properties is visible in the illustration. [Created with BioRender.com.]

Experimental sessions included physical examination, ultrasonography of the AT, high-density surface electromyography (HD-sEMG) of the triceps surae muscles, and torque recordings. A representation of the experimental setup is shown in Fig. 1.

Anthropometric data (age, sex, weight, height, and foot dominance) was obtained. Foot preference in specific daily activities (foot dominance) was determined using a behavioral foot-preference inventory (39). Participants lay prone on the chair of a Biodex System 3 dynamometer (Biodex Medical System), with their knees extended and their tested foot tightly strapped on the footplate. The pelvis was stabilized with another strap to minimize compensatory movements and the ankle was positioned in 0° of plantarflexion with the dynamometer axis aligned with the inferior tip of the lateral malleolus (40). Ultrasonography (LOGIQ S8 GE Healthcare, Milwaukee) was used to confirm the normal structure of the AT. Then, the AT length, thickness, and cross-sectional area were determined during rest (see procedure in *Ultrasonography* section). Afterward, the skin was cleaned and prepared, and the electrodes were placed on the MG, LG, and SO muscles (see details in *Ultrasonography* section). Following the placement of the electrodes, we performed passive elastography assessments. HD-sEMG was used to confirm that the muscles were not active during these measurements as this could influence the estimation of stiffness.

**Figure 2.**
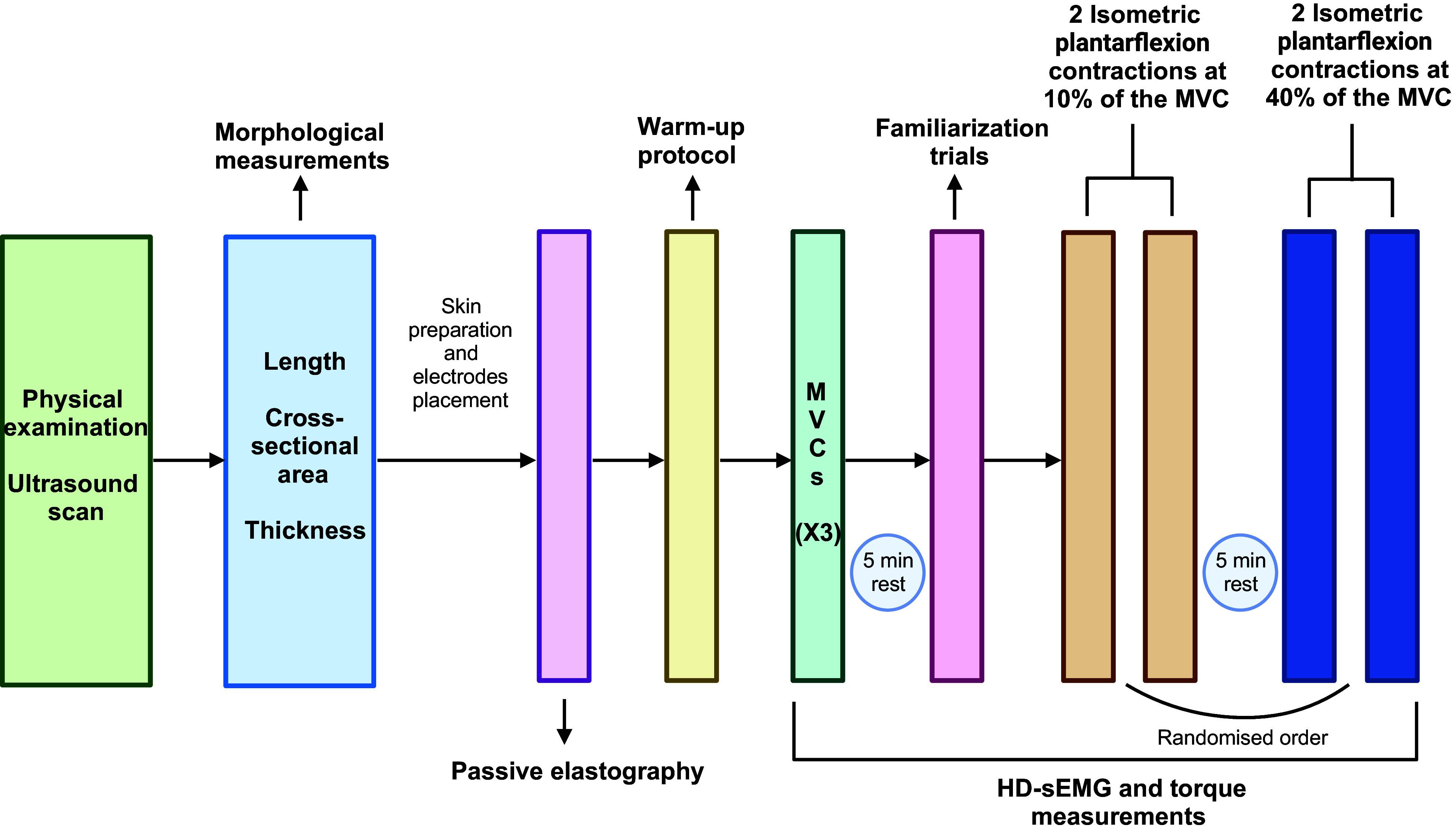
Schematic of the experimental procedure. The order of the contractions performed at each target torque (10% and 40% MVC) was randomized. MVC, maximal voluntary contraction. [Created with BioRender.com.]

Next, participants performed a warm-up protocol consisting of three isometric plantarflexion contractions at their perceived 30% maximal voluntary force for 5 s with 30 s rest between the contractions. Then, the maximal voluntary contraction (MVC) was determined during three isometric plantarflexion contractions (5 s each and 2 min of rest between contractions) (41) at 0° of plantarflexion. The highest MVC value was used as the reference maximal torque. After 5 min rest, participants performed two familiarization trials at 10% and 40% MVC in random order. Following, we measured the activity of the MG, LG, and SO muscles during two isometric plantarflexion contractions at 10%, and 40% MVC (10% MVC/s ramp-up, 10 s hold, 10% MVC/s ramp-down and 30 s rest) with HD-sEMG. The order of the contractions at different target torque levels was randomized using a randomization app (Randomizer) and visual feedback of the target output was provided via a computer monitor positioned 1 m from the participant. A study schematic describing the experimental session is shown in Fig. 2.

### Ultrasonography

All ultrasound images were obtained using an ultrasound imaging device equipped with SWE (LOGIQ S8 GE Healthcare, Milwaukee). All morphological tendon variables (tendon thickness, length, and cross-sectional area) were recorded in B-mode with a 16-linear array probe (50 mm, 4–15 MHz). SWE was recorded in elastography mode with a 9-linear array probe (44 mm, 2–8 MHz).

An adaptation of the protocol developed by Arya and Kulig (42) was used to measure the morphological properties of the AT. Briefly, the ultrasound probe was placed longitudinally over the posterior aspect of the heel, and the calcaneal notch was identified. Then, a fine wire (3.2 × 40 mm) was used under the probe to create an artifact in the ultrasound image. The wire was then aligned with the distal part of the tendon and the corresponding point was marked on the skin with a marker. Then, the ultrasound probe was moved proximally to locate the musculotendinous junction of the MG, and again, a fine wire was used to create an artifact in the ultrasound image. The wire was aligned with the musculotendinous junction of the MG and the corresponding point was marked on the skin. The distance between these two points represented the resting length of the AT. Subsequently, marks were made at 2, 4, and 6 cm above the AT’s insertion, these marks were used as reference to place the middle part of the ultrasound probe in the sagittal plane to determine the thickness of the AT at 2, 4, and 6 cm of its insertion, and three ultrasound images were taken for each mark. Similarly, we used these marks to locate the probe in the transversal plane and obtain the cross-sectional area at 2, 4, and 6 cm of the AT’s insertion, and again three ultrasound images were taken for each mark.

**Figure 3.**
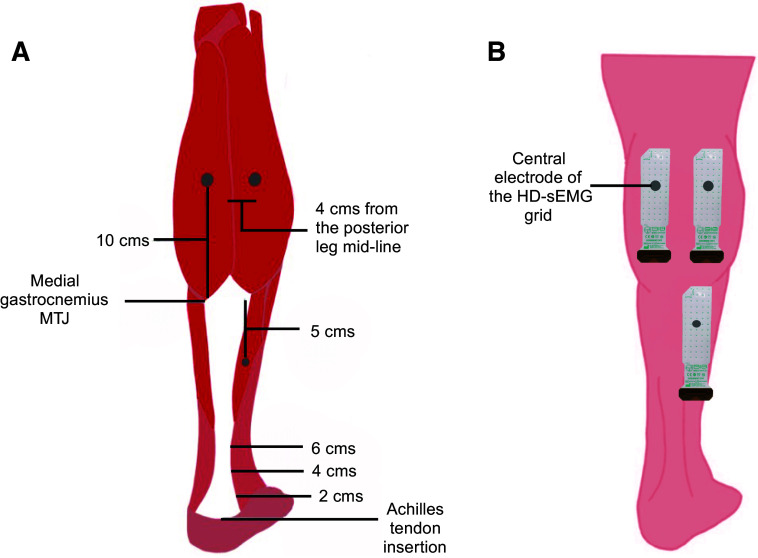
Representation of the anatomical landmarks used for ultrasonography (*A*) and position of the HD-sEMG grids in the MG, LG, and SO muscles (*B*). HD-sEMG, high-density surface electromyography; LG, lateral gastrocnemius; MG, medial gastrocnemius; MTJ, myotendinous junction; SO, soleus. [Created with BioRender.com.]

For the HD-sEMG electrode grid placement, a tape and marker were used to draw a line following the direction of the AT, indicating the mid-line of the posterior leg. For the MG HD-sEMG electrode grid placement, a mark was made 10 cm above the distal musculotendinous junction and 4 cm medial to the mid-line. Similarly, for the LG HD-sEMG electrode grid placement, the leg was marked 10 cm above the distal musculotendinous junction and 4 cm lateral to the mid-line. Likewise, for the SO HD-sEMG electrode grid placement, the leg was marked 5 cm below the distal musculotendinous junction and 4 cm lateral to the mid-line. The central electrodes of the HD-sEMG grids (electrode in row 7 and column 3) were placed on top of all these marks. A representation of the anatomical landmarks used for ultrasonography and electrode placement is shown in Fig. 3.

For the SWE measurements, the ultrasound probe was placed in the sagittal plane, with the middle part of the probe located 4 cm above the AT’s insertion. In addition, a probe holder was used to avoid applying pressure over the tendon and introducing movements that may interfere with the measurements. A test SWE measurement was done to check for possible voids in the estimation and if voids were detected, the ultrasound probe was removed, ultrasound gel was added, and the ultrasound probe was placed again. Passive elastography images were acquired during 12 s (twice). Due to the equipment features, a SWE image was obtained every 2.4 s, thus, to obtain at least four SWE images, the elastography measurements lasted 12 s. Elastography images were checked following each measurement to determine possible voids that may have affected our results.

### Intrarater/Intersession Reliability of the Ultrasonography Measurements

Due to the very low to moderate reliability of the SWE results reported in a recent systematic review (43), we performed an intrarater/intersession reliability analysis of the AT stiffness to check the consistency of these measures. In addition, we performed an intrarater/intersession reliability analysis of the AT morphological properties. Briefly, a group of six participants came to the laboratory for a second experimental session 1 wk apart. Following an identical protocol, the same researcher (ICH) did the SWE measurements on both occasions.

### HD-sEMG and Torque Recordings

HD-sEMG signals were recorded from the MG, LG, and SO muscles using three two-dimensional (2-D) adhesive grids (OT Bioelettronica, Italy) of 13 × 5 equally spaced electrodes (each of 1 mm diameter, with an interelectrode distance of 8 mm) placed in the position described above. The HD-sEMG grid was prepared by attaching a double-side adhesive foam to the grid surface (SPES Medica, Genova, Italy) and by filling the grid cavities with conductive paste, which provided adequate electrode-skin contact (AC-CREAM, SPES Medica, Genova, Italy). In addition, participants’ skin was shaved (if necessary), gently abraded (Nuprep, Skin Prep Gel, Weaver and Company, Aurora, Colorado), and cleaned with water.

All signals were converted from analog-to-digital by a 16-bit analog-digital converter (Quattrocento- OT Bioelecttronica, Torino, Italy). Signals were amplified by a factor of 150, sampled at 2,048 Hz, and filtered with a band-pass filter (bandwidth: 10–500 Hz, first order, −3 dB) (44). HD-sEMG signals were acquired in monopolar mode with ground electrodes (WhiteSensor WS, Ambu A/S, Ballerup, Denmark) positioned in the head of the fibula and with a wet strap in the thigh of the evaluated leg. All the grids and ground electrodes were connected to the same bioelectrical amplifier (Quattrocento-OT-Bioelecttronica, Torino, Italy). The torque exerted by the participants was assessed with a Biodex System 3 dynamometer (Biodex Medical System), which was synchronized with the HD-sEMG signals through the auxiliary input of the EMG amplifier (44).

### Image Analysis

#### Ultrasound image analysis.

After acquiring the ultrasound images, a reference of 1 cm was drawn using the ultrasound tools. Then, the software ImageJ (https://imagej.net) was used to determine the AT thickness at 2, 4, and 6 cm from its insertion. Briefly, the reference was measured with the ImageJ tools, converted into pixels, and set as scale. Then, the length of the image was determined, and the middle point marked on the image. Next, the distance between the superficial and deep part of the paratenon was measured. Afterward, the thickness at 2, 4, and 6 cm was averaged to obtain the AT thickness. Conversely, ultrasound tools were used to determine the CSA of the AT at 2, 4, and 6 cm of its insertion. A discontinuous line was drawn following the internal part of the paratenon as a reference and the CSA was measured. Then, the CSA at 2, 4, and 6 cm was averaged to obtain the AT CSA.

For the SWE measurements, we obtained ∼4 SWE color maps (height × width, 2.5 cm × 1 cm), which were selected using the elastography ultrasound tools to allow a better visualization of the AT. A region of interest (ROI) of 3 mm diameter (45) was selected and located in the middle of the tendon at 4 cm from its insertion to determine the stiffness (kPa). Lastly, mean stiffness was calculated over the ROIs of the four consecutive images recorded (46).

### HD-sEMG Signal Analysis

#### Torque signal analysis.

The highest peak torque exerted during the MVCs (SI: Newton-meters) was used as a measure of maximal plantarflexion strength for each participant (47). The torque signal was low-pass filtered at 15 Hz and then used to quantify the torque steadiness (coefficient of variation of torque, SD torque/mean torque × 100) from the steady phase of the contractions (48). A custom-made MATLAB script was used to plot the torque exerted by each participant, visually identify the steady phase (approximately of 10 s) of the contraction, and select the starting and ending point of the time window needed for the analysis (47).

#### Motor unit analysis.

The HD-sEMG signals recorded during the isometric plantarflexion contractions (10% and 40% MVC) were visually inspected using a custom script created in MATLAB, and the channels with excessive noise were removed (< 5% channels removed). Then, HD-sEMG signals were decomposed into motor unit spike trains with an algorithm based on blind source separation, which provides automatic identification of multiple single motor units (32). Each identified motor unit was assessed for decomposition accuracy with a validated metric (Silhouette, SIL) that represents the accuracy of the decomposed spike train (32), which was set to ≥ 0.90 (49). SIL is a normalized measure of the relative height of the peaks of the decomposed spike trains with respect to the baseline noise (32). The signals were decomposed throughout the whole duration of the submaximal contractions, and the discharge times of the identified motor units were converted into binary spike trains (41).

Discharge times were inspected and edited using a custom-made MATLAB script. Missing pulses producing nonphysiological firing rates (i.e., interspike intervals > 250 ms) were manually and iteratively excluded, and the pulse train was recalculated. In addition, in cases where the algorithm incorrectly assigned two or three pulses for only a single firing, the operator removed this erroneous firing, and the final pulse trains were re-estimated (32). Finally, the mean DR and COV_isi_ were calculated during the steady phase of the torque signal (10 s duration). All single motor unit data were recorded, analyzed, and reported according to the consensus for experimental design in electromyography: single motor unit matrix (50).

#### Motor unit recruitment threshold matching.

Motor unit recruitment threshold was defined as the plantarflexor torque (%MVC) at the time when the motor units began firing action potentials (48). MG, LG, and SO motor units were matched by their recruitment threshold with a tolerance of ±1% MVC. The matched motor units were then grouped into two groups according to their recruitment thresholds (0–10% MVC and 10–40% MVC) (41), to avoid between-muscle differences in recruitment threshold affecting DR and COV_isi_ results, due to potential identification of different populations (low vs. high threshold) of motor units across muscles.

#### Cross-correlation coefficient and neuromechanical delay.

Neuromechanical interactions between motor unit rate coding and force generation were determined using cross-correlation to assess similarities and delays between fluctuations in motor unit firing activity and torque. Delays between the motor unit firing activity and torque were used as a measure of the NMD. Motor unit discharge times obtained were summed to generate a CST that represents the cumulative activity of multiple motor units (32). The signals obtained from CST and torque were smoothed by low-pass filtering (4th order zero-phase Butterworth, 2 Hz) and then high-pass filtering (4th order zero-phase Butterworth, 0.75 Hz) as presented previously (51). Then, filtered CST signals were cross-correlated with torque to determine the similarities in their fluctuations (cross-correlation coefficient) and to obtain the NMD (calculated from the lags found from the cross-correlation function) (32). The cross-correlation coefficient between signals was computed in 5-s segments with 50% overlap (32). The average cross-correlation coefficient and NMD obtained from these segments was reported.

### Statistical Analysis

Descriptive statistics were used to report the data, which are presented as means ± SD, unless otherwise stated. The Shapiro–Wilk test was used to assess data normality. Sphericity was assessed by Mauchly test, and if violated, the Greenhouse-Geisser correction was applied to the degrees of freedom. The level of significance for all statistical procedures was set at *P* < 0.05 and 95% confidence interval (CI) was reported. First, the intrarater/intersession reliability for the morpho-mechanical properties of the AT was assessed. Intraclass correlation coefficient (ICC), a measure of relative reliability, was calculated using a two-way mixed-effects model with absolute agreement. The following criteria were used to determine reliability: <0.5 poor, 0.5–0.75 moderate, 0.75–0.9 good, and >0.9 excellent (52). In addition, the standard error of the measurement (SE) was included as a measure of absolute reliability. The SE represents differences in measurement units, considering both the intervariation within individuals and the variability of the measurement (53), and was obtained from the residual error of a within-subject analysis of variance (ANOVA).

DR and COV_isi_ variables were compared between muscles at each torque level with a linear mixed-model analysis with factors muscle (MG, LG, and SO) and torque (10% and 40%) as fixed effects, and participants as random effect. Cross-correlation coefficients and NMD were compared between muscles and all muscles combined (ALL) at each torque level with a linear mixed model with factors muscle (MG, LG, SO, and ALL) and torque (10% and 40% MVC) as fixed effects, and participants as random effect. For each participant, the DR and COV_isi_ parameters of individual matched motor units were averaged between the two isometric contractions at each torque level for each muscle. These averaged values were then used in the linear mixed model. Similarly, cross-correlation coefficients greater than 0.4 and their corresponding NMD values were averaged between the two isometric contractions at each torque level for each muscle and all muscles combined, resulting in single values that were used in the linear mixed model. We included only muscles/individuals with CST-torque cross-correlation coefficients higher than 0.4 in the analysis, as we observed that cross-correlation coefficients <0.4 provided inaccurate delay/lag (NMD) values (i.e., negative delays). When the linear mixed model was significant, pairwise comparisons were performed with Tuckey post hoc analysis.

A multiple linear regression (stepwise) analysis was performed on the motor unit and motor unit firing-torque relationship parameters to identify the variables that predicted changes in morphological and mechanical variables of the AT. Therefore, morphological and mechanical AT properties (length, thickness, CSA, and stiffness) were used as dependent variables, and motor unit parameters (DR and COV_isi_) and motor unit firing-torque relationships parameters (cross-correlation coefficient between CST and torque, and NMD) were regarded as independent variables. In addition, a multiple linear regression (stepwise) analysis was performed on the motor unit and motor unit firing-torque relationship parameters to identify the variables that predicted changes in torque steadiness. Consequently, torque steadiness was used as a dependent variable and motor unit and motor unit firing-torque relationship parameters were regarded as independent variables.

IBM SPSS Statistics software, V. 29.0 (Armonk, NY), and GraphPad Prism software V.8.0.2 (San Diego, CA) were used for statistical analysis of the data.

## RESULTS

### Intrarater/Intersession Reliability

Intrarater/intersession reliability analysis revealed excellent reliability for length and thickness (ICC: 0.99 and 0.99), good reliability for stiffness (ICC: 0.90), and moderate reliability for CSA (ICC: 0.64) (Table 1).

**Table 1.** Intrarater/intersession reliability results of the ultrasonography morpho-mechanical measures

	ICC (CI)	SEM
Length, cm	0.99 (0.95–0.99)	0.25
Thickness, cm	0.99 (0.97–0.99)	0.002
CSA, cm^2^	0.64 (−0.34 to 0.94)	0.011
Stiffness, kPa	0.90 (0.50–0.99)	2.67

CI, confidence interval; CSA, cross-sectional area; ICC, intraclass correlation coefficient; SEM, standard error of measurements.

### Morphological and Mechanical Properties of the AT

Length, thickness, CSA, and stiffness are presented in Table 2. Means ± SD and minimum-maximum values are reported.

**Table 2.** Morpho-mechanical properties of the AT

	Means ± SD	Minimum-Maximum
Length, cm	19.89 ± 2.57	15.40–25.50
Thickness, cm	0.39 ± 0.04	0.35–0.50
CSA, cm^2^	0.41 ± 0.07	0.31–0.53
Stiffness, kPa	75.95 ± 9.98	53.35–91.66

AT, Achilles tendon; CSA, cross-sectional area; SD, standard deviation.

### Motor Unit Decomposition

Average number of motor units and number of motor units matched by recruitment threshold were reported to illustrate the differences in the number of motor units involved in each analysis. A total of 1,892 motor units were identified in the triceps surae muscle during the submaximal contractions (across all participants). At 10% MVC, the average number of motor units identified was 13.6 ± 17.90, 5.56 ± 9.6, and 12.12 ± 8.95 for the MG, LG, and SO muscles, respectively. At 40% MVC, the average number of motor units identified was 22.44 ± 23.49, 9.64 ± 11.21, and 12.32 ± 7.52 for the MG, LG, and SO muscles, respectively. Regarding the recruitment threshold-matched motor units, a total of 397 motor units were matched between the MG, LG, and SO muscles, with an average of 6.64 ± 7.69 and 9.24 ± 8.94 for each participant during the 10% and 40% MVC tasks, respectively.

### Discharge Rate and Discharge Rate Variability

**Figure 4.**
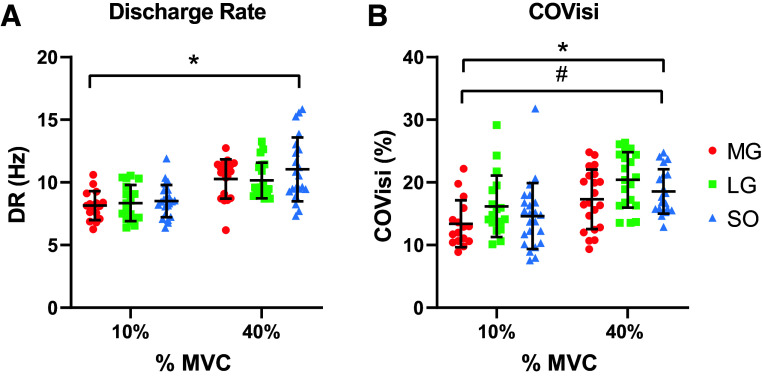
*A*: average motor unit discharge rate (DR) calculated from recruitment-threshold-matched motor units from medial gastrocnemius (MG; red dot), lateral gastrocnemius (LG; green square), and soleus (SO; blue triangle) muscles at 10% and 40% maximal voluntary contraction (MVC). Data points at 10% MVC were MG (*n* = 16), LG (*n* = 18), and SO (*n* = 23); and at 40% MVC were MG (*n* = 21), LG (*n* = 20), and SO (*n* = 21). *B*: coefficient of variation for the interspike interval (COV_isi_) calculated from recruitment-threshold-matched motor units from MG, LG, and SO muscles at 10% and 40% MVC. Data points at 10% MVC were MG (*n* = 16), LG (*n* = 18), and SO (*n* = 23); and at 40% MVC were MG (*n* = 21), LG (*n* = 20), and SO (*n* = 21). A linear mixed model was used for the statistical comparisons. DR and COV_isi_ values (means ± SD) were averaged for each subject and presented at each submaximal target torque (10% and 40% MVC). *Main effect of torque, *P* < 0.0001. #Main effect of muscle, *P* = 0.0069. [Created with BioRender.com.]

DR and COV_isi_ parameters were assessed to investigate the overall motor unit firing rate and variability when motor units were matched by recruitment threshold across muscles. Average DR from MG, LG, and SO muscles at 10% and 40% MVC are presented in Fig. 4*A*. Overall, DR increased as the target torque increased, but DR was similar between muscles (torque effect: *P* < 0.0001, mean difference = −2.11, CI = −2.79 to −1.45, muscle effect: *P* = 0.175). COV_isi_ from the MG, LG, and SO muscles at 10% and 40% MVC are presented in Fig. 4*B*. In general, COV_isi_ increased as the target torque increased, with a difference between muscles; however, no torque-muscle interaction was found (torque effect: *P* < 0.0001, mean difference = −4.00, 95% CI = −5.73 to −2.27; muscle effect: *P* = 0.0069, interaction force-muscle: *P* = 0.90). Given that there were no between-muscle differences in DR, we averaged DR results from all muscles (DR ALL) and inserted this variable into the linear regression. Meanwhile, COV_isi_ results from each muscle were inserted into the multiple regression independently as there were significant differences between muscles.

### Cross-Correlations and Neuromechanical Delay

**Figure 5.**
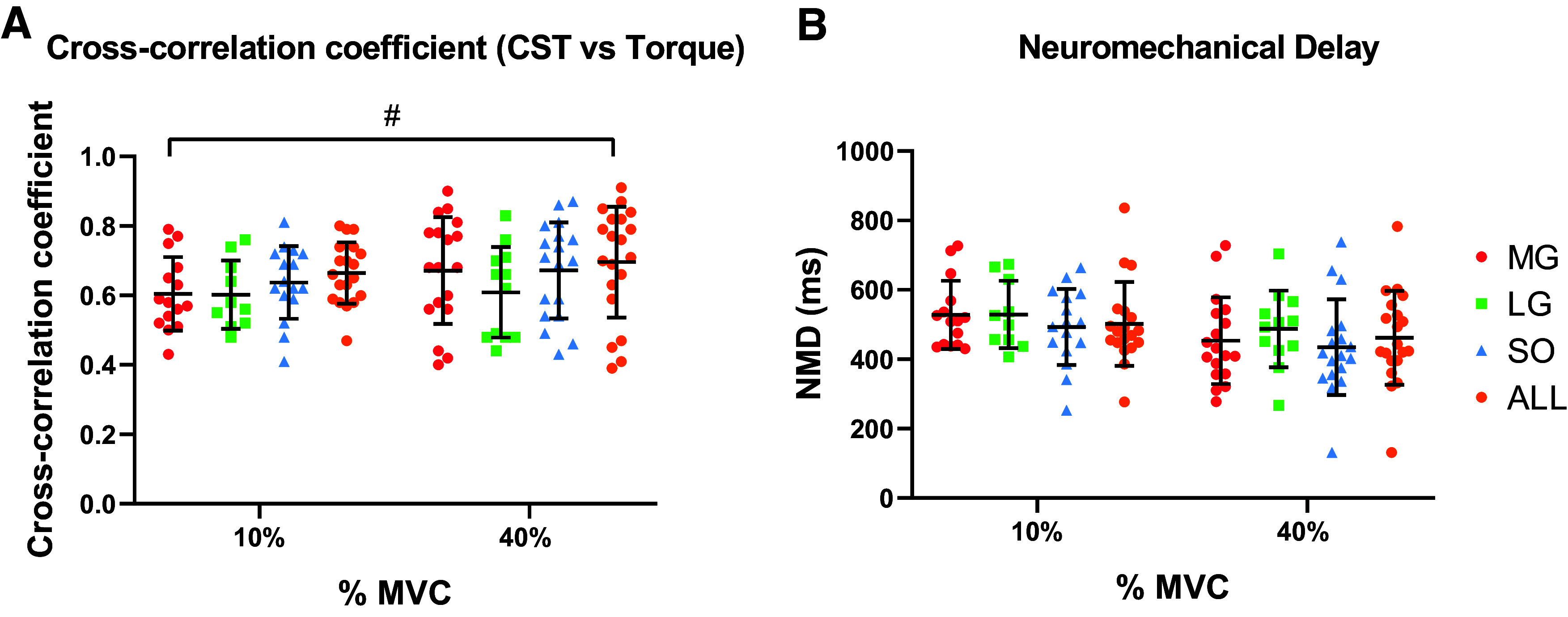
*A*: cross-correlation coefficients between cumulative spike train (CST) versus torque from medial gastrocnemius (MG; red dot), lateral gastrocnemius (LG; green square), soleus (SO; blue triangle), and all muscles combined (ALL: orange dot) at 10% and 40% maximal voluntary contraction (MVC). Data points at 10% MVC were MG (*n* = 15), LG (*n* = 10), SO (*n* = 16), and ALL (*n* = 19); and at 40% MVC were MG (*n* = 18), LG (*n* = 12), SO (*n* = 18), and ALL (*n* = 20). *B*: neuromechanical delay (NMD) from MG, LG, SO, and ALL at 10% and 40% MVC. Data points at 10% MVC were MG (*n* = 15), LG (*n* = 10), SO (*n* = 16), and ALL (*n* = 19); and at 40% MVC were MG (*n* = 18), LG (*n* = 12), SO (*n* = 18), and ALL (*n* = 20). A linear mixed model was used for the statistical comparisons. Cross-correlation coefficient and NMD values (means ± SD) were averaged for each subject and presented at each submaximal target torque (10% and 40% MVC). #Main effect of muscle, *P* = 0.0022. [Created with BioRender.com.]

The cross-correlation coefficient and NMD variables were determined to examine the impact of neural drive generation on force transmission to the tendon. Cross-correlation coefficient between CST and torque from MG, LG, and SO at 10% and 40% MVC are presented in Fig. 5*A*. Overall, cross-correlation coefficients did not change as the target torque increased; however, the cross-correlation coefficient between CST and torque was greater when the CST from all muscles was combined (torque effect: *P* = 0.28, mean difference = −0.034, CI = −0.098 to 0.029, muscle effect: *P* = 0.0022). Furthermore, NMD from MG, LG, and SO at 10% and 40% are presented in Fig. 5*B*. NMD did not change as the target torque increased and did not differ between muscles (torque effect: *P* = 0.06, mean difference 53.69, CI = −279 to 110.2, muscle effect: *P* = 0.73). Therefore, we used the NMD obtained from all muscles (ALL) and we inserted this variable in the multiple regression.

### Multiple Linear Regression

Motor unit variables (DR ALL, COV_isi_ MG, COV_isi_ LG, and COV_isi_ SO) were entered into the multiple linear regression analysis to assess which of these variables was associated with the morpho-mechanical properties of the AT (length, thickness, CSA, and stiffness). Table 3 reports the results of the multiple regressions for these variables.

**Table 3.** Means ± SD and correlation coefficients between dependent variables (morpho-mechanical properties) and independent variables: DR ALL, COV_isi_ MG, COV_isi_ LG, and COV_isi_ SO

Dependent Variable	Means ± SD	Torque Level, %MVC	DR ALL, Hz	COV_isi_ MG, %	COV_isi_ LG, %	COV_isi_ SO, %
Length, cm	20.31 ± 2.80	10	8.23 ± 1.14, *r* = −0.27	12.92 ± 3.88, *r* = −0.31	14.09 ± 2.60, *r* = −0.005	13.43 ± 3.57, *r* = −0.61*
	20.12 ± 2.73	40	10.56 ± 1.52, *r* = −0.20	17.17 ± 4.84, *r* = −0.57*	20.41 ± 4.42, *r* = −0.28	18.28 ± 3.40, *r* = −0.59*
Thickness, cm	0.41 ± 0.04	10	8.23 ± 1.14, *r* = 0.15	12.92 ± 3.88, *r* = −0.22	14.09 ± 2.60, *r* = 0.24	13.43 ± 3.57, *r* = −0.14
	0.40 ± 0.04	40	10.56 ± 1.52, *r* = −0.21	17.17 ± 4.84, *r* = −0.15	20.41 ± 4.42, *r* = −0.05	18.28 ± 3.40, *r* = −0.57*
CSA, cm^2^	0.41 ± 0.07	10	8.23 ± 1.14, *r* = −0.28	12.92 ± 3.88, *r* = −0.44	14.09 ± 2.60, *r* = −0.50	13.43 ± 3.57, *r* = 0.11
	0.40 ± 0.06	40	10.56 ± 1.52, *r* = 0.21	17.17 ± 4.84, *r* = −0.39	20.41 ± 4.42, *r* = −0.27	18.28 ± 3.40, *r* = −0.08
Stiffness, kPa	78.50 ± 8.51	10	8.23 ± 1.14, *r* = −0.69*	12.92 ± 3.88, *r* = −0.65	14.09 ± 2.60, *r* = 0.05	13.43 ± 3.57, *r* = 0.12
	76.03 ± 9.81	40	10.56 ± 1.52, *r* = −0.16	17.17 ± 4.84, *r* = −0.11	20.41 ± 4.42, *r* = −0.10	18.28 ± 3.40, *r* = 0.17

ALL, all muscles; COV_isi,_ coefficient of variation of the interspike interval; CSA, cross-sectional area; DR, discharge rate; LG, lateral gastrocnemius; MG, medial gastrocnemius; %MVC, percentage of the maximal voluntary contraction; SO, soleus. *Significant correlations (*P* < 0.05) are in bold.

When the morphological properties were analyzed as dependent variables, at 10% MVC, only COV_isi_ SO was entered into the model, explaining 30.4% of the variance in the length. However, at 40% MVC, both COV_isi_ MG and COV_isi_ SO were entered into the model, explaining 48.7% of the variance in the length. In addition, at 40% MVC, COV_isi_ SO was entered into the model, explaining 29% of the variance in the thickness.

**Figure 6.**
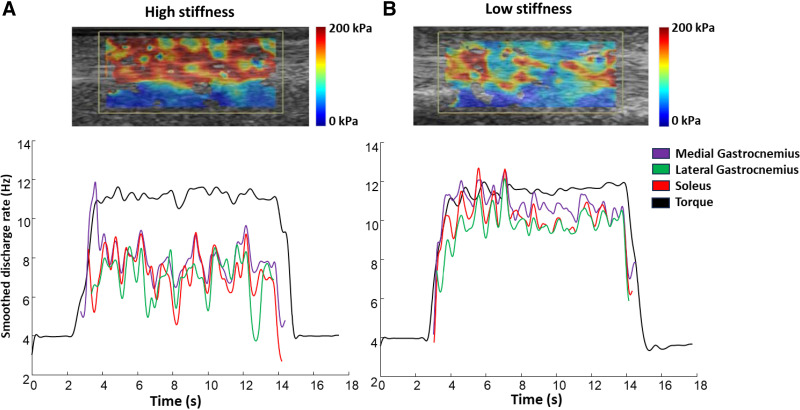
Motor unit discharge rate and Achilles tendon stiffness for two representative participants. *A*: individual with high Achilles tendon stiffness (*top*, shear-wave elastography map) at rest and low discharge rate (*bottom*) for one representative motor unit from the medial gastrocnemius (purple), lateral gastrocnemius (green), and soleus (red) muscles during isometric plantarflexion contractions at 10% maximal voluntary contraction (MVC). *B*: individual with low Achilles tendon stiffness (*top*, shear-wave elastography map) at rest and high discharge rate (*bottom*) for one representative motor unit from the medial gastrocnemius, lateral gastrocnemius, and soleus muscles during isometric plantarflexion contractions at 10% MVC. The black line (bottom) represents the torque exerted by the participants during the contraction. [Created with BioRender.com.]

When tendon stiffness was analyzed as a dependent variable, at 10% MVC, only DR ALL was entered into the model, explaining 41.7% of the variance in AT stiffness. DR ALL was negatively associated with stiffness, meaning that the DR of the triceps surae was higher in tendons with lower stiffness. MG motor unit DR and SWE results for two representative participants with different levels of AT stiffness can be seen in Fig. 6.

Motor unit firing-torque relationship variables (cross-correlation coefficient and NMD) were entered into the multiple linear regression analysis to assess which of these variables were associated with the morpho-mechanical properties of the AT (length, thickness, CSA, and stiffness). When the morpho-mechanical properties were analyzed as dependent variables, none of the motor unit firing-torque relationship variables was entered into the model. Similarly, when we analyzed torque steadiness as the dependent variable, none of the motor unit and motor unit firing-torque relationship variables were entered into the model.

## DISCUSSION

This study revealed that changes in resting tendon stiffness could be predicted by changes in triceps surae motor unit DR at low forces. In addition, motor unit firing rate variability (estimated by COV_isi_) of individual triceps surae muscles was able to predict changes in tendon morphology in a load-dependent manner. Previous studies showed some evidence of the relationship between active/passive tendon mechanics and muscle activity; however, to our knowledge, this is the first study to observe a relationship between motor unit firing and tendon morpho-mechanical properties. By employing HD-sEMG, conventional B-mode ultrasonography, and SWE, we were able to identify neuromechanical relationships relevant for the generation of force.

### Relationship between Motor Unit Firing Rate and Achilles Tendon Morpho-Mechanical Properties

During walking and running, muscular contraction modulates the amount of energy stored in the elastic tissues (9). Therefore, muscles must have the ability to produce or absorb mechanical work, and this behavior is highly dependent on the interactions between the active (myofibrils) and passive (mainly tendon and aponeurosis) elements of the series of elastic components (9, 54). During isometric contractions, there is a shortening of the muscle fascicle and lengthening of the tendon at a fixed ankle joint; therefore, a shortening of the active element produces a lengthening of the passive/elastic element of the series of elastic components (55). In addition, it has been observed that the slackness and compliance of the passive/elastic element allow the fascicle length and pennation angle changes to occur during isometric contractions (55). Furthermore, it has been shown that after a static-stretch intervention of the triceps surae muscles, there is an increase in the DR and a decrease in motor unit recruitment thresholds at low muscle forces, suggesting that the adjustment in motor unit activity is likely related to the change in the compliance of the muscle-tendon unit following stretching (35). Consequently, there is a relationship between DR, tension development, and force-generating capacity (35).

Our results showed that differences in resting AT stiffness could be predicted by changes in the DR ALL at 10% MVC. This is consistent with a previous study, which reported changes in the neural drive of the triceps surae muscles during isometric plantarflexion contractions at 10% MVC but not at 35% MVC after stretching (which increases tendon compliance) (35). This load-dependent relationship between motor unit firing rate and stiffness may be partially explained by the tendon’s mechanical behavior. Tendon stiffness increases at higher muscle contraction levels; however, the rate of change in tendon’s stiffness varies according to the amount of tension placed on the tendon (56). Tendons are lengthened more easily at low forces and then, a plateau in tendon length is reached at higher force levels (55). It can be speculated that individuals with greater tendon compliance at low forces might have required greater motor unit firing output to control the contraction, while at higher forces, the higher tendon stiffness might have allowed a more efficient conversion of neural drive into muscle contraction and then force transmission to the tendon. This greater efficiency in the conversion of neural output into contraction and subsequent transmission of muscle force at higher force levels may have possibly increased the difficulty of detecting variations in tendon stiffness of the participants measured in the current study. Nevertheless, this load-dependent relationship may also be in part explained by the motoneuron modulation that occurs at the motoneuron dendrites and depends on the interactions between descending monoaminergic drive and spinal circuits (57). As Golgi tendon organ mechanoreceptors can detect rapid changes in contractile force (58), and Ib afferents mostly inhibit homonymous motoneurons through di/tri-synaptic connections (59), it can be hypothesized that tendons with reduced stiffness might decrease the sensitivity of the Golgi tendon organ, lessening the inhibitory input to the α-motoneuron and therefore explaining the increase in the DR in tendons with greater compliance.

In addition, multiple regression analysis results showed that changes in length could be predicted by changes in COV_isi_ SO at 10% MVC; however, changes in length could be predicted by changes in COV_isi_ MG and COV_isi_ SO at 40% MVC. These results might be explained by differences in the contribution of each of the triceps surae muscles to the net force at different target torques. In support of this notion, a recent study reported that during isometric plantarflexion contractions at 10% MVC, the activation ratio of the SO muscle was higher than the activation ratio of the MG, and both were higher than the activation ratio of the LG. However, at 50% MVC, the activation ratio of the SO decreased to similar values of the activation ratio of the MG, and both were higher than the activation ratio of the LG (60), suggesting that the COV_isi_ of both muscles was able to predict changes in the AT’s length probably because at 40% MVC, they have a similar level of activation. However, we also observed that changes in the AT thickness could be predicted by changes in the COV_isi_ SO at 40% MVC, indicating that these relationships between the variability of the DR and the morphological properties of the tendon are not just influenced by the level of activation of each individual muscle, it might be possible that the DR modulation of each muscle transmits differently to the tendon, even when the resultant modulation (torque steadiness) seems to be not affected by this, as we did not find associations between COV_isi_ and torque steadiness.

### Neural Drive to MG, LG, and SO Muscles

It is difficult to compare the neural drive (motor unit DR and recruitment) received by agonist muscles due to the limitations in HD-sEMG decomposition techniques that are only able to identify a subset of the populations of active motor units (41). However, an indirect assessment of the neural drive received by synergistic muscles can be estimated by comparing firing parameters from motor units matched by recruitment threshold (41). This approach minimizes the effect of recruitment threshold-dependent variations in DR between muscles, which can be due to the identification of different populations of motor units by the decomposition algorithm. By employing this approach, we observed no differences in motor unit DR between muscles at 10% and 40% MVC (Fig. 4*A*). Nevertheless, when we estimated the motor unit firing rate variability through COV_isi_, we observed differences between muscles. Specifically, the COV_isi_ was higher in the LG compared with the MG at 40% MVC (Fig. 4*B*). This is an interesting finding as a recent study has shown minimal common drive between MG and LG muscles during isometric plantarflexion contractions estimated by coherence analysis between CST of each muscle at similar target torques (61). The relative independent control of these muscles may allow for flexible control of the ankle joint to comply with their functions (e.g., maintaining balance, joint stabilization, distribution of tendon strain) during different tasks (61). This theory is partially supported by studies showing smaller volume and longer fascicles in the LG muscle compared with the MG (60), and different actions in the frontal plane (62–64).

Another method to estimate the effective neural drive to muscles is to sum the spike trains of the involved motor units and then smooth the resultant signal to produce a continuous estimate of the command signal (CST) (65). Our findings indicate that CST was moderately correlated with fluctuations in the isometric plantarflexion torque at 10% and 40% MVC (R = 0.67 and R = 0.69, respectively). The strength of our cross-correlation coefficients results was higher compared with the one reported by Mazzo et al. (65), in the triceps surae muscles at 10% (R = 0.582) and 35% (R = 0.612). However, the strength of the cross-correlation coefficients between the CST estimates and torque fluctuations for the triceps surae muscle was not as strong as the one observed in tasks where a single muscle is involved, likely due to the differences in the experimental protocol (65). For example, Thompson et al. (66) found higher cross-correlations between the neural drive and torque in isolated SO muscles during evoked contractions (R = 0.84). Even so, this difference may be explained by the different species assessed (human vs. cat) (67) or type of contraction (voluntary vs. electrically evoked). Other possible explanations are the involvement of other muscles (e.g., intrinsic foot muscles or accessory lower leg muscles) during the plantarflexion tasks (65) or the variable level of activation among the triceps surae muscles without compromising the net force (68, 69). In addition, our results showed no differences in the cross-correlation coefficients between the MG, LG, and SO at 10% and 40% MVC, the only difference observed was between MG and ALL at 10% MVC (Fig. 5*A*). Moreover, no differences were observed as the target torque increased. These results indicate the cross-correlation coefficient between the CST and torque of the MG, LG, and SO can be used separately to estimate the moment-to-moment fluctuations in force during isometric plantarflexion contractions at low- and moderate-target torques. For this reason, we used the average delay quantified from the cross-correlation function from all muscles to calculate the NMD described below.

### Neuromechanical Delay

The conversion of neural signals to force output has a latency due to the dynamic sensitivity of the motor neurons and the time needed to stretch the series of elastic components of the muscle-tendon unit following the electrical activation of the muscle fibers (70). Previous studies have used the electromechanical delay to determine the time lapse between the onset of muscle electrical activation and onset of force/torque production (71–76). However, this method does not provide information on the delay between neural drive to muscle and force (70). Due to this reason, in our study, we used the NMD, which is defined as the time difference between the neural drive and the generated force/torque during a voluntary contraction. The NMD can be estimated from the time lag of the peak of the cross-correlation between the CST and torque (70). Our results showed that the mean estimated NMD was 502.05 ± 120.48 ms and 461.80 ± 135.25 ms at 10% and 40%, respectively. Overall, our results showed higher NMD values compared with previous studies. Del Vecchio et al. (69) and Martinez-Valdes et al. (51) reported NMD values of ∼300 ms in the tibialis anterior muscle during isometric dorsiflexion contractions modulated at low frequencies and low target torques (32). These differences may be explained by the different muscles assessed (tibialis anterior vs. triceps surae), the different contractions evaluated (modulated vs. not modulated), or the dynamometer used. In addition, our results showed no difference in the NMD between muscles at 10% and 40% MVC, neither as the target torque increase (Fig. 5*B*), which is in agreement with the findings from Martinez-Valdes et al. (32).

### Implications for Future Research

Our findings demonstrate a contraction-intensity-dependent relationship between motor unit firing parameters of the triceps surae muscle and the morpho-mechanical properties of the AT. However, the remaining variance in the morpho-mechanical parameters can likely be attributed to a combination of multiple factors. Motor unit-tendon interactions may have been influenced by muscle force transmission to connective tissue (77), sampling of the pool of active motor units, and changes in muscle contractile properties (78). In addition, other factors such as individual differences (e.g., age, sex, and genetic predisposition), physical activity level, and measurement factors (e.g., position of the ankle during the measurement, probe pressure over the tendon, cross talk from other muscles, and identification of superficial motor units) could have also played a role. Therefore, future studies aiming to generate predicting models of muscle-tendon interactions should consider these factors for an accurate estimation.

## METHODOLOGICAL CONSIDERATIONS

There are some methodological aspects of this study that should be considered. First, intrarater/intersession reliability analysis of the AT morpho-mechanical properties was performed on only six participants. This analysis aimed to evaluate the researcher’s consistency in assessing AT morpho-mechanical properties. Previous studies have demonstrated that ultrasonography provides good to excellent reliability in determining the morphological properties of the AT (21), while SWE has shown moderate reliability in assessing the AT’s mechanical properties (45). Second, the study lacks a familiarization session, which may have improved the execution of the isometric plantarflexion contractions. Nevertheless, familiarization trials were conducted before the isometric plantarflexion contractions to instruct the participants on how to perform the tasks. Third, the ankle attachment of the isokinetic dynamometer used had two lever arms to measure plantarflexion; thus, the time to detect the torque may have been longer, influencing the results of the NMD. Finally, current ultrasound imaging devices with SWE have very limited sampling resolution (0.5–2 SWE images per second); future developments in SWE technology might enable improved and concurrent assessment of the interplay between tendon stiffness and motor unit firing properties.

### Conclusions

This study shows a contraction-intensity-dependent relationship between the motor unit firing parameters of the triceps surae muscle and the morpho-mechanical properties of the AT. The most relevant finding is that individuals with increased resting tendon stiffness showed lower DR at low target torque force. This novel approach provides valuable insights into the complex neuromechanical interactions during low-force voluntary isometric tasks. Our research contributes to a more comprehensive understanding of the underlying mechanisms involved in neural coding and muscle-tendon unit behavior and how this interplay impacts force generation during such tasks.

## DATA AVAILABILITY

The data and analysis codes are available from the corresponding author, EM-V, upon reasonable request.

## GRANTS

This work was supported by ANID PhD Scholarship awarded by the Government of Chile. Recipient: Ignacio Contreras-Hernandez, Scholarship ID number: 72200295. F. Negro was funded by the European Research Council Consolidator Grant INcEPTION (Contract No. 101045605).

## DISCLOSURES

No conflicts of interest, financial or otherwise, are declared by the authors.

## AUTHOR CONTRIBUTIONS

I.C.-H., D.F., and E.M.-V. conceived and designed research; I.C.-H and M.A. performed experiments; I.C.-H. and E.M.-V. analyzed data; I.C.-H. and E.M.-V. interpreted results of experiments; I.C.-H. prepared figures; I.C.-H. drafted manuscript; I.C.-H., M.A., D.F., F.N., and E.M.-V. edited and revised manuscript; I.C.-H., M.A., D.F., F.N., and E.M.-V. approved final version of manuscript.
